# 肺段切除术中段间平面显示方法的研究进展

**DOI:** 10.3779/j.issn.1009-3419.2020.101.31

**Published:** 2020-09-20

**Authors:** 亮 陈, 明建 葛

**Affiliations:** 400016 重庆，重庆医科大学附属第一医院胸外科 Department of Thoracic Surgery, The First Affiliated Hospital of Chongqing Medical University, Chongqing 400016, China

**Keywords:** 肺段切除术, 段间平面, 显示, Segmentectomy, Intersegmental plane, Identification

## Abstract

近年来随着计算机断层扫描（computed tomography, CT）的普及，早期肺癌被大量发现，肺段切除术得到广泛临床应用。段间平面的显示是肺段切除术的关键步骤之一，当前显示段间平面的方法众多且各有优劣，我们将对相关方法进行回顾，以期对临床实践有所帮助。

## Abstract (en)

With the popularity of computed tomography (CT) scan in recent years, early stage lung cancer has been discovered in large numbers of patients and pulmonary segmentectomy has been widely used clinically. Identification of the intersegmental plane is one of the key steps in pulmonary segmentectomy, and current methods for identifying the intersegmental plane are numerous and have their own advantages and disadvantages. We will review relevant methods to help the clinical practice.

随着高分辨电子计算机断层扫描（high-resolution computed tomography, HRCT）在肺癌筛查中不断普及，被检出肺磨玻璃结节（ground glass opacity, GGO）的患者越来越多^[1]^。出于减少手术创伤和保护肺功能的目的，肺段切除术在临床上逐渐得到广泛应用^[2, 3]^。大量研究证明肺段切除术较肺叶切除术远期生存效果相当，但由于保留了更多的肺组织，患者术后生活质量更高^[4-8]^。段间平面的识别是肺段切除术的关键步骤之一^[9]^，不准确的段间平面识别将可能导致剩余肺组织无功能、通气或血流不匹配，甚至术后长期漏气需要二次手术^[10]^。目前段间平面显示技术根据原理可分为三类：通气差异、循环差异和支气管内染色剂。本文对PubMed数据库内2000年-2020年1月，含有关键词“intersegmental plane”和“segmentectomy”的文献进行检索，通过阅读进行筛选，同时对重要文献的参考文献进行扩展阅读，现将对十余种方法现状进行综述（表 1）。

**1:** 节间平面形成技术 Techniques of intersegmental plane formation

Author	Method	Preparatory work (target segment)			Target segment	Reserved segment	Disadvantage
		Brochnus	Artery	Vein			
Tusbota^[10]^	Inflation-deflation method	Isolated			Inflation	Deflation	Not applicable for emphysema patients, narrow field of VATS vision
Oizumi^[19]^	Slip-knot bronchial ligation	Isolated			Inflation	Deflation	Not applicable for patients with emphysema or airway obstruction
Matsuoka^[22]^ Okada^[23]^	Selective target-segment bronchial ventilation with bronchoscopy	Isolated			Inflation	Deflation	Additional equipment and worker
Kamiyoshihara^[24]^	Butterfly needle injection	Isolated			Inflation	Deflation	Risk of air embolism, difficulty in injection
Wang^[26]^	Modified inflation-deflation method	Cut	Cut	Cut	Inflation	Deflation	Need for adequate 3D CT, positive pressure ventilation, Postoperative pain
Misaki^[10]^	ICG injection in systemic vein		Cut		White	Blue	Short duration, additional equipment
Sugimoto^[38]^	Indigo carmine injection in target-segment artery		Cut		Blue	Red	Difficulty in injection, not yet used clinically
Iwata^[40]^	Target segment pulmonary arteriovenous occlusion		Cut	Cut	Inflation	Deflation	Need for adequate 3D CT
Sakamoto^[41]^	Thermal imaging method		Cut	Cut	Light red	Crimson	Extra surgical incision, not yet used clinically
Sekine^[30]^	ICG injection in target-segment bronchus with bronchoscopy	Injection			White	Grey	Requires additional equipment and worker, risk of ICG reflux, risk of inaccurate dispersion
Zhang^[27]^	Methylene blue in target-Segment bronchus	Cut	Cut	Cut	Blue	Red	Risk of inaccurate dispersion
Oh^[20]^	ICG injection in target-segment bronchus	Cut		Cut	Green	Red	Risk of ICG exceed the intersegmental plane
Waseda^[34]^	Vitamin B2 injection in target-segment bronchus with bronchoscopy	Cut			Yellow-green	Red	Additional equipment, not yet used clinically
Elkhouly^[44]^	ICG injection in reserved segment bronchus with bronchoscopy	Cut	Cut	Cut	Blue	White	Additional equipment and worker, risk of inaccurate dispersion
CT: computed tomography; VATS: video-assisted thoracoscopic surgery; ICG: indocyanine green.							

## 1 段间平面的表现

段间平面或称段间隔膜（intersegmental septum）是两个或多个肺段之间的一层薄膜，是脏层胸膜表面向内的延伸^[11, 12]^，电子显微镜下段间平面是由三层结构组成，表面两层由与肺泡相邻的肺泡壁组成，肺泡壁上皮细胞通过紧密连接相连，可防止物质进出，中间一层为胶原纤维^[13]^，其中包含每段对应的段间静脉和淋巴管^[10]^，CT上可能观察到部分段间平面，具体原理尚不清楚，有研究^[14]^认为可能与存在段间静脉、肺间质水肿和肺泡内蛋白沉积等原因有关。

## 2 根据通气差异显示段间平面

2000年Tusbota等^[15]^提出膨胀-萎陷法，该法利用靶段充气和保留段放气确定膨胀-萎陷界限。其优点是能准确识别段间平面所在位置且操作方便。相较于胸外科教程所提到的传统方法^[16]^，膨胀萎陷法未使用正压通气，避免了Kohn’s孔的打开，能准确识别段间平面。但该方法不能识别肺实质内的段间平面，肺气肿患者段间平面显示效果欠佳，术者将受充气的肺组织影响其胸腔镜视野^[17, 18]^。

基于这一原理，2014年Oizumi等^[19]^提出靶段支气管滑结结扎法，在分离靶段支气管后做Roeder结并于肺组织充气后拉紧，线结闭合靶段支气管管腔使得靶段充气，保留段萎陷，从而形成膨胀-萎陷线。该方法相较于Tusbota等^[15]^的方法仅改善了受充气的肺组织影响视野这一不足^[15, 17, 20, 21]^。Matsuoka^[22]^和Okada^[23]^于2003年及2007年先后提出经纤支镜靶段支气管通气的肺段切除术，采用胸腔镜辅助小切口途径（hybrid video-assisted thoracoscopic surgery）进胸，由麻醉医师使用3.5 mm柔性纤维支气管镜于靶段支气管高频喷气（频率40 Hz，压力2 kg/cm^2^），靶段膨胀后将靶段支气管栓线结扎使得靶段持续充气，保留段萎陷，从而形成膨胀-萎陷线。该方法可以快速识别段间平面，且选择性靶段通气对胸腔镜视野视野影响较小，但靶段支气管定位并不简单，需要有经验麻醉医师配合，且针对不同直径的肺段支气管或亚肺段支气管，纤支镜直径均需要调整。Kamiyoshihara等^[24]^于2007年提出蝴蝶针靶段支气管充气法，分离出靶段支气管后直视下将蝴蝶针刺入靶段支气管进行通气。该方法和Matsuoka^[22]^和Okada^[23]^提出的方法相似，省去了纤支镜通气这一步骤，但存在刺入肺动脉导致空气栓塞可能。目前有1例病例报道提到使用蝴蝶针靶段支气管充气法导致空气栓塞最终导致患者死亡^[25]^。Wang等^[26]^于2017年提出改良膨胀-萎陷法，用切割缝合器或结扎的方式处理靶段支气管、靶段动脉和段内静脉后，以20 cm H_2_O的压力患侧正压通气，气体通过Kohn’s孔进入靶段内，待患侧完全膨胀，再单通健侧，患侧与大气相通。因靶段内气体无法通过肺循环或气道流失，约5 min-12 min可在膨胀的靶段和萎陷的保留段之间形成膨胀-萎陷线，以此显示段间平面。该方法不需要额外设备，节省了打结的步骤，减少了充气肺组织对胸腔镜视野的影响。但存在依赖3D-CT、术后疼痛和需要正压通气等不足。有学者认为该方法需进行充分的术前准备，术中需保留段间静脉，离断段内静脉，充分等待段间平面完全显示，若错误切断或保留血管、等待时间不足都将导致段间平面显示不准确^[10, 18, 26]^；膨胀的靶段肺组织在取出时由于体积过大，可能会挤压胸腔镜伤口，导致术后疼痛严重^[18, 26]^；术中需要麻醉师膨肺，可能存在正压通气伤^[18]^，Zuo等^[13]^的研究表明未发现段间平面存在Kohn’s孔的证据，并提出正压通气可能会挤破段间平面隔膜这一假设。

综上所述，通过膨胀-萎陷线确定段间平面这类方法不需要使用染剂，使用设备较少，Tusbota^[15]^、Okada^[23]^和Wang^[26]^的方法在临床应用较多。但此类方法对术者普遍要求较高，如使用Tusbota^[15]^、Oizumi^[19]^和Okada^[23]^的方法需先分离靶段支气管，而靶段支气管处于肺组织深部，单独分离显示且对其远心断端腔内注气并非易事，尤其是在分离过程中误伤血管时，暴露靶段支气管将更为困难；Wang等^[26]^提出的方法需要完全离断靶段内所有段门结构，但在术中判断静脉走形和归属并非易事，即使使用3D-CT也无法完全保证术中的精准识别；Matsuoka^[22]^和Okada^[23]^提出的方法需要麻醉医师配合使用喷气式纤支镜。Kamiyoshihara等^[24]^提出的方法安全性还有争议。

## 3 根据循环差异显示段间平面

2009年Misaki等^[10]^首次提出将吲哚菁绿（indocyanine green, ICG）应用于肺段切除术段间平面的识别中，离断靶段动脉后经外周静脉注射ICG溶液（3.0 mg/kg），使用的红外线胸腔镜（infrared thoracoscopy, IRT）发射805 nm和940 nm两种红外光，含有ICG的保留肺组织吸收805 nm的光，反射940 nm的光，在IRT下呈蓝色。而靶段不含ICG，呈白色，以此显示段间平面。该方法可用于肺气肿患者，避免了膨肺的视野影响。但存在ICG过敏风险、需要额外设备、染色持续时间较短等不足。多项研究表明使用ICG不能适用于肝功能差、ICG过敏或碘过敏的患者^[17, 27, 28]^。ICG的毒性反应与浓度相关，当浓度超过5 mg/kg，过敏反应频率显著增加^[29]^，而未经稀释的ICG具有明确的组织毒性，可引起炎症、溃疡和上皮组织损伤^[30-32]^；红外胸腔镜需额外购买且价格不菲；ICG在肺循环内存在时间较短，仅能在肺表面标记出界限，且复杂肺段切除需要大量或重复使用ICG，多次使用ICG可能导致ICG溶液通过支气管循环进入靶段肺组织，同时也加重了组织毒性风险^[17, 20, 33, 34]^。有学者提出临时夹闭整个肺静脉以延长染色持续时间，但又产生了阻断肺循环形成血栓的风险^[33, 35]^。Kasai^[36]^和Kuroda^[37]^在识别光谱数量和浅表毛细血管成像方式（Spectra-A系统）上提出改进方法，均使成像效果和染色持续时间得以提升。

基于这一原理，2011年Sugimoto^[38]^提出靶段肺动脉内注射靛蓝胭脂红（indigo carmine）的肺段切除术，结扎靶段动脉后向远心端注射靛蓝胭脂红使靶段蓝染。相较于Misaki等^[10]^提出方法，靛蓝胭脂红是一种更加安全的临床常用染色剂，并且不需要使用IRT系统，但该方法尚未运用于临床，且染色效果尚未得以证实。有研究指出该方法段间平面准确性可能不如经支气管染色^[30]^，实际操作难度较高^[39]^。2012年Iwata等^[40]^提出靶段肺动-静脉阻断的肺段切除术，切除靶段动静脉后纯氧膨肺，待完全膨胀后健侧通气。此时靶段由于肺循环被阻断无法转运氧气，故处于持续膨胀状态，而保留段内氧气经肺循环流失而萎陷，以此显示膨胀-萎陷线。相较于前两者，该方法不需要额外设备和染剂，且未使用正压通气，可用于更复杂的肺段或亚肺段切除术，理论上该方法和Wang等提出的改良膨胀-萎陷法同样需要克服段内静脉和段间静脉识别的问题，且在靶段支气管尚存在的情况下，要离断所有靶段肺血管在技术上难度较大。2016年Sakamoto等^[41]^提出热成像辅助的肺段切除术，离断靶段动脉后靶段和保留段因血流差异产生将温度差异，使用热成像仪扫描以显示段间平面，但该方法尚未运用于临床，缺乏后续研究，理论上该方法不需要膨肺或染色，但需要扩大手术切口以便热成像仪扫描。

综上所述，通过循环差异显示段间平面需要术前排除靶段肺血管变异以保证段间平面准确显示。Misaki等^[10]^提出的方法在临床应用较多，相关研究丰富，但受限于需额外使用设备和染色持续时间。Iwata等^[40]^所提出的方法优点明显，使用成本低，方法便捷，但缺乏后续研究。Sugimoto^[38]^和Sakamoto^[41]^提出的方法尚处于动物实验阶段，还需要进一步研究。

## 4 靶段支气管内注入染色剂显示段间平面

2012年Sekine等^[30]^提出红外胸腔镜辅助纤支镜支气管内注射ICG的肺段切除术，该方法经纤支镜向靶段内冲入20 mL-30 mL浓度为5 mg/mL的ICG溶液，再将200 mL-300 mL气体注入靶段支气管内以分散ICG，通过红外胸腔镜可观察到靶段和保留段之间的段间平面，该方法对肺气肿患者有效，相较Misaki等^[10]^提出的方法，该方法经支气管确定靶段区域，便于联合肺段或亚肺段切除，缺点是需要使用IRT系统，需要麻醉医师纤支镜配合，存在造影剂反流可能，并且支气管内的染色剂分布将受到支气管内痰液影响。

Oh等^[20]^于2013年提出直视下靶段支气管注射ICG的肺段切除术，该方法在结扎靶段静脉和支气管后直视下用静脉输液套管缓慢将50 mL的ICG溶液（0.5 mg/mL）注入远端靶段支气管内。相较于Sekine等^[30]^的方法，此法不需要购买IRT系统，但需提前精准切断段内静脉，手动注射ICG，注射快慢将影响ICG分布效果，注射过快将导致ICG通过段间平面的Kohn’s孔向保留段扩散^[18]^。2013年Waseda等^[34]^提出PDD内镜辅助下靶段支气管注射VB2的肺段切除术，切断靶段支气管后直视下将VB2溶液注入远端靶段支气管内，使用PDD内镜可观察到段间平面，该方法优点在于持续时间更长，染剂安全。但该方法尚未运用于临床，缺乏后续研究。2015年Zhang等^[27]^提出靶段支气管内注射亚甲蓝的方法，切断靶段动静脉和支气管后直视下将亚甲蓝溶液注入远端靶段支气管中，亚甲蓝是常用且安全的染色剂，肺表面和实质均被染色，不需要特殊设备且适用于肺气肿或肝功能差的患者。大量研究^[42, 43]^证明亚甲蓝广泛运用于临床检查和治疗。但靶段支气管内的染色剂分布将受到支气管阻塞或痰液影响。2018年Elkhouly等^[44]^提出红外胸腔镜辅助纤支镜下保留段支气管注射ICG的肺段切除术，切断靶段动静脉和支气管后将ICG溶液通过纤支镜注射入保留段支气管中，该方法不会造影剂反流，然而需要与麻醉医师配合，染剂分布受支气管内痰液影响。

综上所述，经靶段支气管染色是准确的，通过术前HRCT或三维CT支气管血管成像（three-dimensional computed tomography bronchography and angiography, 3D-CTBA）即可判断是否存在支气管变异，但需注意注射染剂之前需充分吸尽支气管内痰液。Sekine^[30]^和Elkhouly^[44]^提出的方法较其他方法步骤更多，而Zhang^[27]^和Oh^[20]^提出的方法更为便捷，不需要额外设备和人员。

## 5 小结与展望

肺段切除术目前广泛应用于胸外科临床，段间平面显示技术是肺段切除术的关键步骤之一，也是困难环节。以上十余种段间平面显示技术虽优缺点明显，但不可否认，技术的迭代更新均向着安全、有效且简单的方向发展，那些在手术步骤、不需要额外设备或染剂和成像效果占优势的技术必将淘汰处于劣势的技术，我们相信在未来将会发明出对术者更友好、对患者更经济、对手术更有效的段间平面显示技术。
